# Supplementary material for: Asymmetry in kinematic generalization between visual and passive lead-in movements are consistent with a forward model in the sensorimotor system
Source: PLoS One. 2020 Jan 29;15(1):e0228083. doi: 10.1371/journal.pone.0228083 (PMC6988934; doi:10.1371/journal.pone.0228083)
Supplement: Attachment — Submitted filename: Rebuttal Round 2.docx [file pone.0228083.s002.docx]

Response to the Reviewers

We thank both of the reviewers for their concerns and suggestions and we have addressed these issues in the current version.

Reviewer #1: The authors have addressed most of my concerns.

I somewhat regret that they pushed back more than they attempted to verify their claim: one of my concerns was that should participants be able to learn that distinct kinematics are followed by channels, then perhaps the force applied to the channel wall decreased not because there was no generalization, but because participants knew there would be no force field. It is not an unreasonable objection, and it does not take anything away from the interesting finding that the visual system seems to operate differently. That some effect already happened in the beginning based on one block was not very convincing. I recommend that the authors either leave that option open and even discuss why they don’t think it could happen, or better, check it with a design in which generalization trials are not systematically followed by force channels.

We disagree with the reviewer’s concerns here for several reasons. First of all, even experienced participants (which are not being tested here in our study, since participants were naïve) are normally unable to detect which trials are a channel trial and which contain a force field. Perhaps the reviewer may not have had experience with this set-up and experimental design which may lead to this confusion. Secondly, the reviewer states that they do not find the fact that we see our effects on the very first block of channel trials (a single channel trial for this condition) convincing. This is *the most* convincing result. The participants have no way of knowing before this point, that these test trials have a channel trial (instead of a curl field) following this slight variation in the preceding visual or passive motion. That is, there is no possible way for them to know a priori that there would not be a force field here. Even more important, participants can’t even detect after the trial has finished whether this was a channel trial or a force field trial, since the forces they experience in the two conditions are the same. The suggestion from the reviewer – either including a force field or having a null field – cannot be adopted to probe generalization as it would completely affect the results either by introducing learning or unlearning respectively. We have many more points to support our case, but perhaps the most relevant to this reviewer is that if this occurred as the reviewer suggests, then it would result in not the smooth variation in generalization forces on the channel but either force being produced or not being produced – i.e. a discrete rather than continuous effect on the generalization as the reviewer initially suggested and which we already discuss and outline why this is not the case within our discussion. We have added to the discussion outlining our position as requested.

Reviewer #2: The authors have addressed most of the previous comments. There are just a few points that still remain.

1) Of course there are previous studies that have used only 8 subjects to show reproducible effects, but the question is if 8 subjects is sufficient for the current study? At six trials per condition/subject (for 34 conditions) this is a reasonable question.

There are several important points to make here. First of all, the reviewer is mistaken regarding the number of generalization trials. We have 16 repeated measurements for every channel trial condition. This means that each single point on our generalization measure is made up of the combination of 128 independent measurements. Moreover, our design does not require a single difference at any one of the various channel trials, but instead investigates the overall differences in the generalization function, whereby each generalization description is now made of 128 x 14 = 1792 trials. Here, eight participants provide an extremely strong and robust effect, which can easily be seen in our statistics. Particularly, all three comparisons of interest (visual generalization, passive generalization, and comparison of the two) produce main effect differences in our ANOVAs of p<0.001. Again, the critical point is that our results do not depend on any specific comparison of the 14 different conditions (although statistically speaking these are very strong as well), but explores the overall effects of varying kinematics.

While perhaps a slightly orthogonal point, we believe that it is also important to point out that in our design that we do not simply record participants performing a few movements in 20 minutes of activity and then depend on statistics to detect a difference. Instead, by designing an experiment where we continue to measure participants behaviour over 6 hours of recording we are able to record sufficient data to see effects in a single participant. Our current manuscript is made up of 96 hours of individual movements from our participants.

2) Did the authors actually mean 235 here and throughout the revision?:
“The 0o target location was associated with lead-in start locations at +135o and 235o, and the 270o target location was associated with lead-in start locations at +45o and 135o.”

We thank the reviewer for pointing this out. We have corrected it to 225°.

3) Figure 2 could be clarified if the coordinate system was included with respect to the movements depicted.

We have now indicated the movement directions in the legend of Figure 2.

4) For completeness it would be nice (but not critical) for the authors to show/state that the asymptotic training level of force compensation between passive and visual lead in groups in Figure 3 are not significantly different.

Although this comparison is not the point of our study, we now include this test. As expected from our figures, we found a significant difference between the asymptote of the two groups of participants using a t-test – and have added this result to the methods section.
